# An endogenous protein inhibitor, YjhX (TopAI), for topoisomerase I from *Escherichia coli*

**DOI:** 10.1093/nar/gkv1197

**Published:** 2015-11-08

**Authors:** Yoshihiro Yamaguchi, Masayori Inouye

**Affiliations:** 1 The Osaka City University Advanced Research Institute for Natural Science and Technology (OCARINA), 3-3-138 Sugimoto, Sumiyoshi-ku, Osaka 558-8585, Japan; 2 Faculty of Biology, Graduate School of Science, Osaka City University, 3-3-138 Sugimoto, Sumiyoshi-ku, Osaka 558-8585, Japan; 3 Department of Biochemistry and Molecular Biology, Center for Advanced Biotechnology and Medicine, Rutgers-Robert Wood Johnson Medical School, Piscataway, NJ 08854, USA

## Abstract

Almost all free-living bacteria contain toxin-antitoxin (TA) systems on their genomes and the targets of toxins are highly diverse. Here, we found a novel, previously unidentified TA system in *Escherichia coli* named *yjhX-yjhQ*. Induction of YjhX (85 amino acid residues) causes cell-growth arrest resulting in cell death, while YjhQ (181 residues) co-induction resumes cell growth. The primary cellular target of YjhX was found to be topoisomerase I (TopA), inhibiting both DNA replication and RNA synthesis. Notably, YjhX has no homology to any other toxins of the TA systems. YjhX was expressed well with an N-terminal protein S (PrS) tag in soluble forms. PrS-YjhX specifically interacts with the N-terminal region of TopA (TopA67) but not full-TopA in the absence of plasmid DNA, while PrS-YjhX binds to full-TopA in the presence of DNA. Notably, YjhX does not directly interact with DNA and RNA. YjhX inhibits only topoisomerase I but not topoisomerase III and IV *in vitro*. Hence, *yjhX* is renamed as the gene for the TopA inhibitor (the *topAI* gene). TopAI is the first endogenous protein inhibitor specific for topoisomerase I.

## INTRODUCTION

Almost all free-living bacteria contain a number of toxin–antitoxin (TA) operons, and these toxins target various cellular functions to regulate cell growth and death. The toxins and their cognate antitoxins are co-expressed from a toxin-antitoxin (TA) operon (1,2). These TA systems are classified into five groups (types I–V) on the basis of nature and function of the antitoxin (3). The most studied TA system among them is the type II TA system, in which antitoxins consist of proteins that inhibit the function of their cognate toxins by forming a stable TA complex. However, under stress conditions, stress-induced proteases degrade unstable antitoxins to release toxins from the TA complexes. The *Escherichia coli* K12 genome encodes at least 33 putative TA systems, which, according to biochemical and bioinformatic analyses, consist of type I and type II systems (1). The first TA system discovered was a plasmid-borne type II system that has a role in plasmid maintenance known as post-segregational killing (4). These TA systems are not essential for normal cell growth, although they are widely conserved in bacteria and archaea (1,5). Interestingly, pathogens such as *Mycobacterium tuberculosis* possess a large number of the TA systems (88 TA systems) in comparison with their non-pathogenic counterpart, *M. smegmatis* (2 TA systems) (5), suggesting that the TA systems may play an important role in bacterial pathogenicity. The mechanisms for the toxin actions are highly diverse depending upon cellular targets of the toxins.

Deciphering the cellular target and inhibitory function of each toxin is important for understanding the roles of the TA systems in bacteria. All type I toxins function as the inhibitors of ATP production, whereas the cellular targets of the toxins of the type II TA systems are highly diverse. Known cellular targets of toxins of the type II toxins include the ATP synthesis, DNA gyrase, mRNA, rRNA, tRNA, 30S ribosomes, 50S ribosomes and cytoskeleton proteins (1). Among the cellular targets for the *E. coli* TA system toxins, mRNA is the most common target, perhaps because inhibition of mRNA function is the mildest means of inhibiting cell growth. Out of the 33 known or predicted TA system toxins, 11 are known to interfere with mRNA function. As all of these 11 toxins cleave cellular mRNAs, they are termed mRNA interferases or RNA restriction enzymes.

In bacteria, there are four main DNA topoisomerases: topoisomerase I, DNA gyrase, topoisomerase III and topoisomerase IV (6). Topoisomerase I and III are type IA topoisomerases that interact with single-stranded DNA, while DNA gyrase and topoisomerase IV are type IIA topoisomerases that interact with double-stranded DNA (7). Although these four topoisomerases have different functions during a cell cycle, the DNA supercoiling level is primarily set by opposing actions of DNA topoisomerase I and gyrase (6). Topoisomerase I relaxes negatively supercoiled DNA and is required to prevent the chromosomal DNA from becoming extensively negatively supercoiled, while topoisomerase III appears to be optimized for resolving replication and recombination intermediates (8–11). It was shown that topoisomerase I relaxes negative supercoiled DNA more efficiently than topoisomerase III (12). DNA gyrase consisting of GyrA and GyrB introduces negative supercoils into DNA. Topoisomerase IV participates in chromosome decatenation and relaxes both positive and negative supercoiled DNA (13). DNA gyrase and topoisomerase IV are good targets for antibiotics since these enzymes are essential for bacterial cell growth, their inhibition leads to cell death and their structures are different from those of the mammalian enzymes (14–16). However, there are few reports about the antibiotics targeting bacterial topoisomerase I (17–19). Inactivation of topoisomerase I (TopA) results in the production of hypernegative supercoiled plasmid DNA *in vivo* (6,7). It has been shown that deletion of *topA* is lethal (20–22). However, it has also been reported that a *topA* deletion mutant is viable in the presence of topoisomerase III (*topB*) (23,24) or compensatory mutations usually found in DNA gyrase (25). Therefore it is still unclear if *topA* is a truly essential gene in *E. coli*.

During our search for the TA systems on the *E. coli* genome, the *yjhX-yjhQ* operon appears to be a potential TA system due to the small sizes of the genes (85 amino acid residues for YjhX and 181 residues for YjhQ) and the fact that their respective open reading frames are separated by eleven base-pairs. In this paper, we demonstrate that YjhX-YjhQ is a new *E. coli* TA system consisting of a toxin, YjhX and an antitoxin, YjhQ. Notably, YjhX has no homology to any other toxins. This toxin inhibits topoisomerase I activity but not topoisomerase III and IV *in vitro*. These results indicate that YjhX is the first protein inhibitor specific for topoisomerase I. Therefore, we renamed YjhX as *top*oisomerase I *i*nhibitor gene (*topAI*). Our data also indicate that TopA is an essential gene in *E. coli*

## MATERIALS AND METHODS

### Bacterial strains and plasmids

*E. coli* BL21(DE3) and DH5α were used. *E. coli* PLK831 [F-, *gal-25*, *λ^−^*, *ΔtrpE63*, *pyrF287*, *fnr-1*, *IN(rrnD-rrnE)1*, *rpsL195*(strR), *iclR7*(Const), *trpR72*(Am)] and RS2 [F-, *gal-25*, *λ^−^*, *topA10*, *pyrF287*, *fnr-1*, *rpsL195*(strR), *iclR7*(Const), *trpR72*(Am)] were provided from the Coli Genetic Stock Center. The *topAI* gene was amplified by polymerase chain reaction (PCR) using the *E. coli* genomic DNA as template and cloned into pET28a (Novagen). The *yjhQ* gene was also amplified and cloned into pBAD24 (26) and pCold-PST (27). The gene encoding only the N-terminal domain of Protein S (PrS) was amplified with use of pCold-SPSTI_N_ as template (28) and cloned into pET28a-*topAI* to express PrS-TopAI fusion protein. The *topB*, *topA* and truncated mutants, *topA67* and *topA14* were also amplified by PCR and cloned into pET28a to express the N-terminal His-tagged TopB, TopA, TopA67 (67 kDa; residues 1–597) and TopA14 (14 kDa; residues 745–865), respectively. A Strep-tag (WSHPQFEK) (29) was added to PrS_2_-*yjhQ* by PCR using the primer to construct pCold-PST-*yjhQ-stp* containing only the Strep-tag at the C-terminal end.

### Assay of DNA replication, RNA and protein synthesis *in vivo*

A 10 ml culture of *E. coli* BL21(DE3) containing pET-*topAI* plasmid was grown at 37°C in M9 medium supplemented with glucose. When the O.D._600_ of the culture reached 0.3, 1.5 ml of the culture was transferred to a tube containing 20 μl [^3^H]thymidine (Perkin Elmer) and 80 μl cold thymidine (1 mg/ml) for DNA synthesis or 20 μl [^3^H]uridine (Perkin Elmer) and 80 μl cold uridine (1 mg/ml) for RNA synthesis, respectively. A 50-μl aliquot of the culture was spotted on a 3MM filter paper (Whatman 3 mm, 2.3 cm diameter) at indicated times and the filter paper was soaked in 10% TCA, which was then incubated for 1 h at room temperature. Then the filter papers were washed three times with 10% TCA solution. The filter papers were analyzed using a scintillation counter. Protein synthesis was analyzed as described previously (30). Data are representative of three independent experiments.

### Protein purification

pET-PrS-*topAI*, pCold-PST-*topAI*, pET-*topB*, pET-*topA*, pET-*topA67* and pET-*topA14* were introduced into *E. coli* BL21(DE3) to purify N-terminal His-tagged PrS-TopAI, PrS_2_-TopAI, TopB, TopA, TopA67 and TopA14, respectively. The expression of these proteins was induced with 1 mM isopropyl-β-D-1-thiogalactoside (IPTG) for 3 h at 37°C. The His-tagged proteins were purified with use of Ni-NTA agarose (Qiagen) following the manufacture's protocol. PrS-YjhQ-Stp was also expressed as described above and purified with Strep-tactin (Qiagen) following the manufacture's protocol.

### RNA isolation and Northern blotting analysis

*E. coli* BL21(DE3) cells containing pET-*topAI* were grown at 37°C in M9-glucose medium. When the O.D._600_ value reached 0.4, IPTG was added to a final concentration of 0.1 mM. The samples were taken at different intervals as indicated in Figure 2E. Total RNA was isolated using the hot-phenol method as described previously (31). Northern blot analysis was carried out as described previously (30).

### Analysis of DNA topology *in vivo*

The *E. coli* BL21(DE3) carrying pET-*topAI* was cultured in M9-glucose at 37°C. When O.D._600_ of the culture reached 0.6, different amounts of IPTG were added as indicated in Figure 3. A 20-ml culture was taken at indicated times and centrifuged with 5000 r.p.m. at 4°C for 10 min. The pET-*topAI* plasmid was extracted from the collected cells by using NucleoSpin Plasmid kit (Clontech) and 800 ng of plasmid DNA was analyzed by electrophoresis on 1.0% agarose gel containing 2.5 μg/ml chloroquine (Sigma) in Tris-borate EDTA (TBE) buffer. The gel was run for 12 h at 3 V/cm at room temperature, washed with water for 2 h and stained with ethidium bromide. The DNA bands were then visualized by UV trans-illumination.

### Analysis of DNA topoisomerase I, III and IV activities

Supercoiled pUC19 plasmid DNA (0.6 μg) was incubated with 50 nM DNA topoisomerase I (New England Biolab) for 30 min at 37°C in the presence of different amounts of PrS-TopAI in a 20-μl reaction buffer containing 20 mM Tris-acetate (pH 7.5), 50 mM potassium acetate, 10 mM magnesium acetate, 1 mM DTT and 1 μg/ml BSA. The reaction was stopped by the addition of 2 μl of 0.1 mM EDTA or 2.5 μl of 5% SDS and 2.5 μl of proteinase K (3 mg/ml). The samples were analyzed by 1.0% agarose gel electrophoresis in the presence or absence of chloroquine (40 μg/ml). Purified topoisomerase III (TopB) was incubated with pUC19 plasmid DNA in reaction buffer similar to that used for measuring of topoisomerase I activity as described above. Topoisomerase IV (TopoGEN) was incubated with pUC19 in 40 mM Hepes-KOH buffer (pH 8.0) containing 100 mM potassium glutamate, 10 mM magnesium acetate, 10 mM DTT, 20 mM ATP and BSA (50 ug/ml) in the presence and absence of TopAI. The reaction was stopped by the addition of 2 μl of 0.1 mM EDTA and the samples were analyzed as described above.

### Immunoprecipitation assay

PrS-TopAI or PrS_2_ and TopA, TopA67 or TopA14 at a molar ratio of 1:5 were incubated in 300 μl of buffer A [20 mM Tris-HCl (pH 8.0) and 150 mM NaCl] in the presence or absence of 1 μg/ml pUC19 plasmid for 30 min at 4°C, and then 10 μl of anti-protein S antiserum was added to the reaction mixtures. After incubation for 2 h at 4°C, protein G agarose (Roche) was added and the mixture was incubated for 1 h at 4°C. The protein bound agarose was collected by centrifugation, washed six times with the buffer A containing 0.1% TritonX-100 and then dissolved in 50 μl of 2×SDS-PAGE sample applying buffer (SAB) containing (120 mM Tris-HCl, pH 6.8, 4% SDS, 150 mM NaCl, 20% glycerol and 0.001% bromophenol blue). The samples (10 μl) were subjected onto a 12.5% SDS-PAGE, followed by western blot analysis using 6xHis mAb/HRP conjugate (Clontech).

### Pull-down assay

PrS-TopAI and PrS containing only a His-tag were purified using Ni-NTA and PrS_2_-YjhQ-Stp containing only Strep-tag was purified with Strep-tactin (Qiagen) following the manufacture's protocol. The PrS_2_-YjhQ-stp was incubated with PrS-TopAI and PrS in buffer A at 4°C for 30 min, respectively. Ten microliters of Ni-NTA resin were added to each mixture and the final mixtures were incubated for another 1 h. The resin containing the complexes was washed three times with 1 ml of buffer A containing 0.1% Triton X-100 and re-suspended in 50 μl of 1× SAB for 10 min, and the suspended mixture was kept in a boiling water bath for 5 min. The samples were separated on a 12.5% SDS-PAGE, and visualized by western blot analysis using anti-His antibody (Clontech) and anti-NWSHPQFEK antibody (Genscript), respectively.

## RESULTS

### TopAI and YjhQ is a TA system

**Figure 1.**
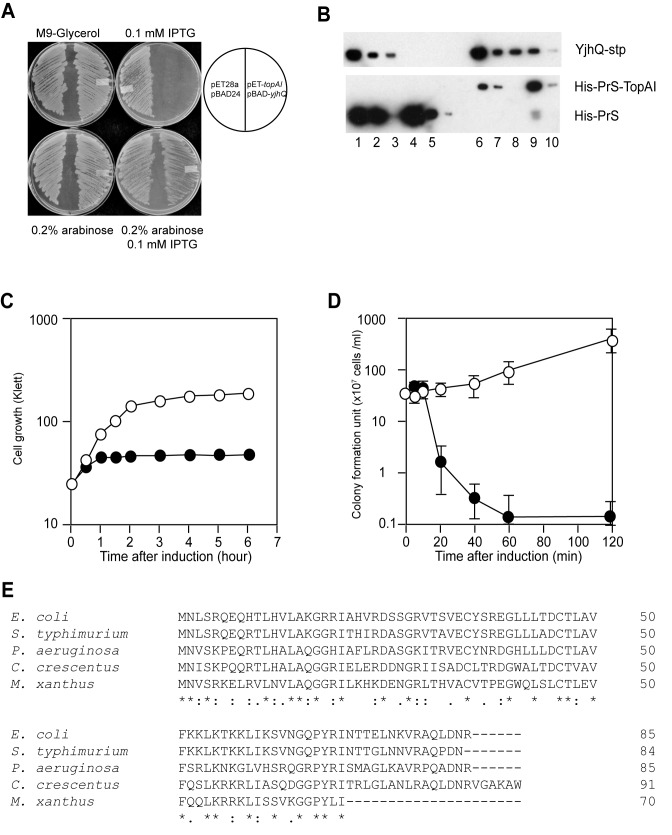
Identification of TopAI and YjhQ as a TA system. (**A**) *E. coli* BL21 transformed with pET28a and pBAD24 or pET-*topAI* and pBAD-*yjhQ* was streaked on M9 (glycerol, CAA) plates with 0.1 mM IPTG, 0.2% arabinose, 0.1 mM IPTG plus 0.2% arabinose or without both inducers. The plates were incubated at 37°C for 18 h. (**B**) Interaction between TopAI and YjhQ in a pull-down assay. Purified PrS-TopAI or PrS containing a His-tag and PrS_2_-YjhQ containing Strep-tag were incubated at 4°C for 1 h. The complex was recovered by nickel-resin. The mixture (lanes 1 and 6), flow-through (lanes 2 and 7), wash fraction (lanes 3 and 8) and elution fraction (lanes 4, 5 and 9, 10) were analyzed by western blotting using His-tag antibody or Strep-tag antibody. (**C**) Growth curves of *E. coli* BL21(DE3) harboring pET-*topAI*. The cells were cultured in M9-glucose liquid medium at 37°C in the presence (closed circles) or absence (open circles) of 0.1 mM IPTG. (**D**) Colony formation units after induction of TopAI. *E. coli* BL21(DE3) harboring pET-*topAI* was cultured in M9-glucose. When O.D. reached 0.6, 0.1 mM IPTG was added. The cells were collected, washed three times with saline and spread on M9-glucose plates. The plates were incubated at 37°C for 18 h and the number of colony was counted. (**E**) Alignment of *E. coli* TopAI with other TopAI homologues from *Salmonella typhimurium*, *Pseudomonas aeruginosa*, *Caulobacter crescentus* and *Myxococcus xanthus*. Identical amino acid residues are shown in black shades and conservative substitutions in gray shades.

The YjhQ and TopAI genes were cloned into an arabinose inducible pBAD24 plasmid (26) and an IPTG inducible pET28a plasmid, respectively. *E. coli* BL21(DE3) cells harboring both pBAD-*yjhQ* and pET-*topAI* could not form colonies on M9-glycerol-casamino acids agar plates in the presence of IPTG (0.1 mM) (Figure 1A). However, co-induction of YjhQ in the presence of 0.2% arabinose with TopAI in the presence of 0.1 mM IPTG neutralized the toxicity of TopAI indicating that the overproduction of TopAI is toxic to the cells, while YjhQ is the antitoxin for TopAI. Since toxins usually form an oligomeric complex with cognate antitoxins (1), we decided to analyze the interaction between TopAI and YjhQ by a pull-down assay. However, we observed that TopAI and YjhQ cannot be expressed as soluble proteins. Thus, we created fusion proteins for both TopAI and YjhQ with Protein S (PrS), a major Ca^2+^ binding spore coat protein from *Myxococcus xanthus* (27). PrS-tag fusion has been shown to significantly enhance the solubility and expression of several proteins and is very useful for protein purification (27,32). To distinguish TopAI and YjhQ, we created a construct in which the N-terminal domain of Protein S is fused to TopAI (PrS-TopAI; 20 kDa) and also a tandemly repeated Protein S tag is attached to YjhQ (PrS_2_-YjhQ; 42 kDa). PrS-TopAI and PrS_2_-YjhQ were highly expressed in soluble form in *E. coli*. Notably, PrS-TopAI induction caused cell growth arrest as seen with induction of TopAI by itself, indicating that PrS has no effect on TopAI toxicity. The purified PrS protein was used as control. His-tagged PrS or PrS-TopAI was incubated at 4°C for 1 h with PrS_2_-YjhQ-Stp which also contained a Strep-tag (29) in addition to a PrS_2_ tag. Then Ni-NTA agarose was added to the reaction mixtures and the agarose-protein complex thus formed was collected by centrifugation. The agarose was washed six times with Tris-HCl buffer (pH 7.4) containing 150 mM NaCl and 0.1% TritonX-100, and suspended in SDS-PAGE loading buffer. As shown in Figure 1A, PrS_2_-YjhQ was pulled down with PrS-TopAI (Figure 1B; lanes 9 and 10) but not with PrS (Figure 1B; lanes 4 and 5), indicating that TopAI toxin interacts with YjhQ antitoxin in a manner similar to that of other TA systems. We concluded that TopAI and YjhQ are toxin and antitoxin, respectively in a novel TA system in *E. coli*.

### Effect of TopAI on cell growth

Next, we examined the toxicity of TopAI in a liquid culture (Figure 1C). Induction of TopAI using pET-*topAI* plasmid in *E. coli* completely inhibited cell growth after 60 min induction and dramatically reduced colony forming units (0.16%) even at 30 min after induction, indicating that TopAI is a bactericidal toxin (Figure 1D). BLAST search showed that TopAI is conserved in *Salmonella*, *Caulobacter, Pseudomonas* and *Myxococcus* but not in gram-positive bacteria, archaea and eukaryotes (Figure 1E). These TopAI homologs have 53%–59% identity and 70%–74% homology to *E. coli* TopAI.

### TopAI inhibits DNA and RNA synthesis

**Figure 2.**
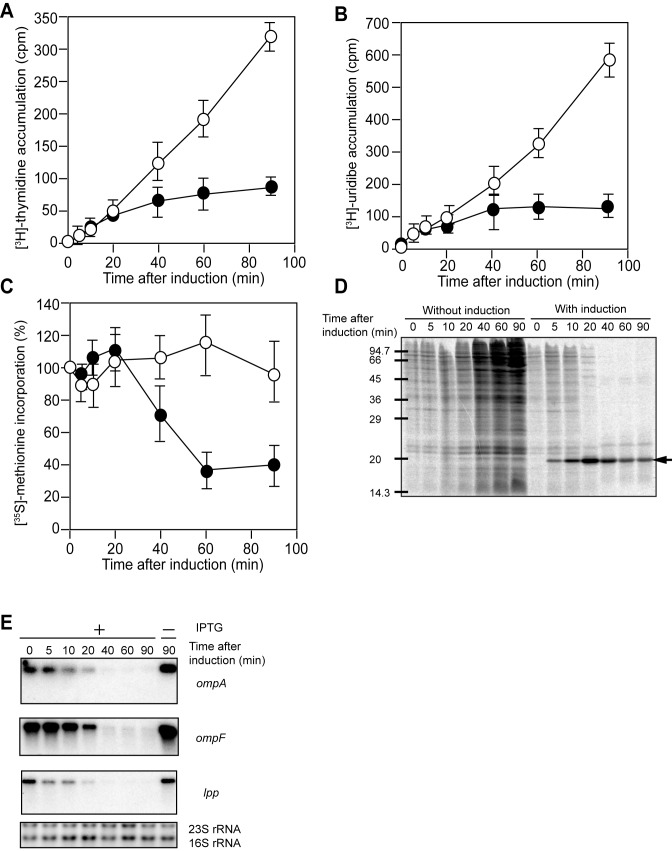
Effect of TopAI on DNA, RNA and protein synthesis. Effect of TopAI on [^3^H]thymidine and [^3^H]uridine accumulation *in vivo* (**A** and **B**). (**C**) Effect of TopAI on [^35^S]methionine incorporation *in vivo*. At the different time intervals indicated, protein synthesis was analyzed as described previously (53). (**D**) SDS-PAGE analysis of the products from C. The reaction mixture at the time points indicated were were collected. The samples were applied to 15% SDS-PAGE gel. (**E**) Effect of TopAI on cellular mRNA stability. Total RNA was extracted from *E. coli* BL21(DE3) cells harboring pET-*topAI* at various time points as indicated after the addition of IPTG (0.1 mM) and subjected to northern blotting with labeled *ompA*, *ompF* and *lpp* as probes, respectively. Before transferring RNA onto the membrane, the gel was stained with ethidium bromide to detect 23S rRNA and 16S rRNA.

To identify the target(s) of TopAI, we next examined if DNA replication, transcription and translation were inhibited by TopAI. Analysis of the TopAI overexpressing cells was carried out as described previously (33). [^3^H]thymidine and [^3^H]uridine incorporation was completely inhibited at 40 min after TopAI induction (Figure 2A and B). However, 40% [^35^S]methionine incorporation was still achieved at 90 min (Figure 2C) and the newly synthesized protein bands were detected at 90 min after induction of TopAI (Figure 2D). This indicates that the target of TopAI may be DNA replication and/or RNA synthesis but not protein synthesis. Northern blot analysis after TopAI induction showed that the full-length *ompA, ompF* and *lpp* mRNAs gradually disappeared without generating shorter cleavage products (Figure 2E), indicating that TopAI inhibits RNA synthesis but does not function as an endoribonuclease.

### TopAI accumulates excess supercoiled plasmid DNA

**Figure 3.**
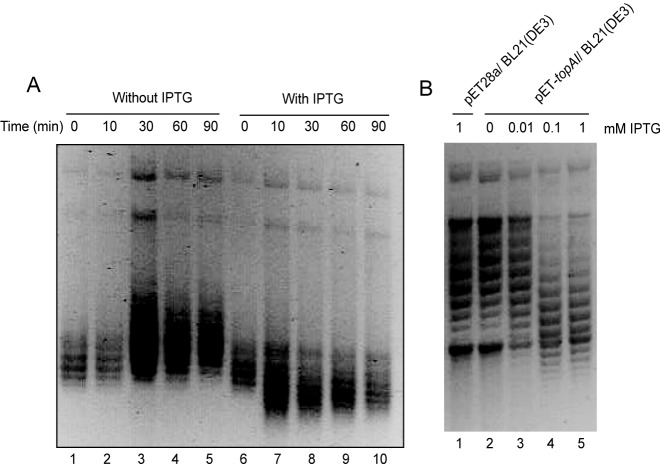
Effect of TopAI on DNA topology. (**A**) *E. coli* BL21(DE3) harboring pET28a or pET-*topAI* was grown at 37°C in M9-glucose liquid medium. When O.D._600_ reached at 0.6, IPTG was added to a final concentration of 0.1 mM. At the different time intervals indicated, 50 ml of the culture were removed and DNA topology of plasmid was analyzed. These analyses were carried out in the presence of 2.5 μg/ml chloroquine. (**B**) *E. coli* BL21(DE3) harboring pET-*topAI* was grown at 37°C in M9-glucose liquid medium. When O.D._600_ reached at 0.6, different concentration of IPTG was added (lane 2; 0 mM, lane 3; 0.01 mM, lane 4; 0.1 mM and lane 5; 1 mM). After 1 h incubation, 50 ml of the culture were removed and DNA topology of the plasmids was analyzed as described in Materials and Methods. *E. coli* BL21(DE3) containing pET28a was incubated with 1 mM IPTG and the extracted plasmid was analyzed as negative control (lane 1).

In *E*. *coli*, two enzymes involved in DNA replication and transcription by virtue of their influence on DNA topology are DNA gyrase (promotes supercoiling) and topoisomerase I (promotes relaxation of DNA) (6). We thus next tested if either of these enzymes were inhibited by TopAI by analyzing the effect of overexpression of TopAI on DNA topology. We analyzed plasmids from cells overexpressing TopAI at the time intervals shown in Figure 3A in the presence of 2.5 μg/ml chloroquine. Under these conditions, compact DNA migrates faster allowing better resolution of supercoiled DNA (34). Ten minutes after induction of TopAI, an excess amount of supercoiled plasmid appeared (Figure 3A, lane 7). However, pET28a extracted from cells incubated with 0.1 mM IPTG as control showed the same electrophoretic pattern at each time point. These data indicate that TopAI inhibits topoisomerase I and not DNA gyrase. We also analyzed plasmids from cells overexpressing TopAI in different concentrations of IPTG as shown in Figure 3B. An excess amount of supercoiled plasmid appeared in the presence of 0.1 and 1 mM IPTG (Figure 3B, lanes 4 and 5). However, pET28a extracted from cells incubated in the presence of 1 mM IPTG as control showed same electrophoretic pattern in the absence of IPTG (Figure 3B, lanes 1 and 2). Based on these data, we concluded that the observed inhibition of both DNA replication and RNA synthesis after induction of TopAI is due to the inhibition of topoisomerase I activity.

### TopAI inhibits topoisomerase I activity i*n vitro*

**Figure 4.**
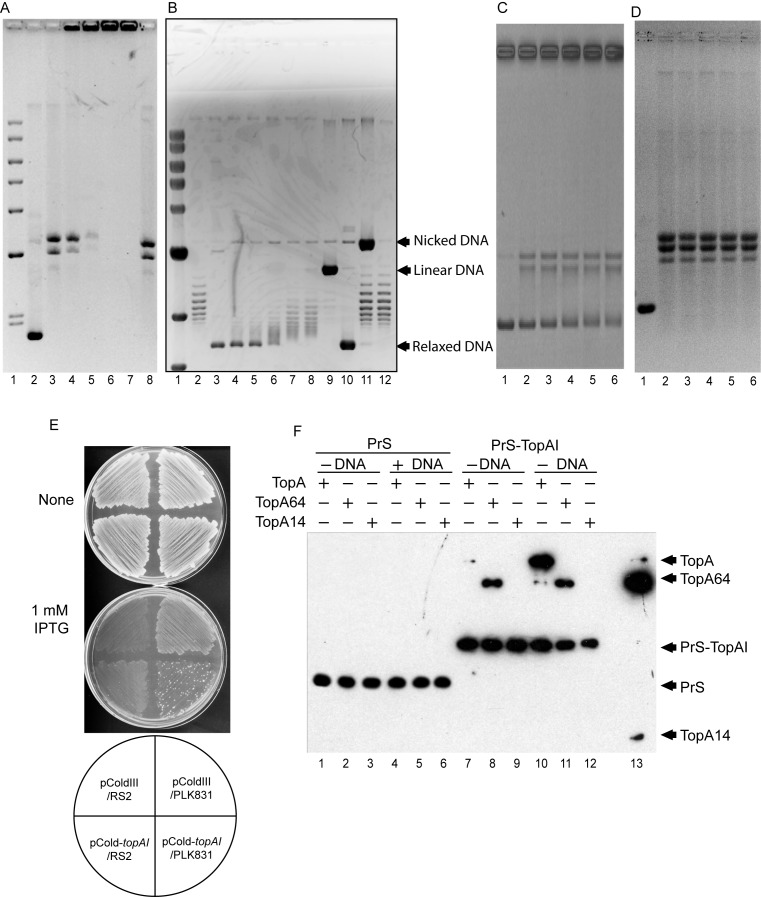
Effect of TopAI on the topoisomerase activity. (**A**) DNA relaxation assay with 50 nM TopA was carried out using pUC19 plasmid as substrate with agarose gel electrophoresis in the absence of chloroquine. *Lane 1*, DNA molecular weight markers, *lane 2*, without TopA; *lane 3*, without PrS-TopAI; *lanes 4–7*, 20, 50, 100, and 200 nM PrS-TopAI, respectively; *lanes 8* 200 nM PrS-TopAI plus 200 nM PrS-YjhQ. The reaction was stopped by the addition of EDTA. (**B**) DNA relaxation assay with agarose gel electrophoresis in the presence of chloroquine. *Lane 1*, DNA molecular weight markers, *lane 2*, without TopA; *lane 3*, without PrS-TopAI; *lanes 4–8*, 10, 20, 50, 100, and 200 nM PrS-TopAI, respectively; lane 9–12, linear, relaxed nicked and supercoiled pUC19 DNA. The reaction was stopped by the addition of proteinase K and SDS. (**C** and **D**) Effect of TopAI on topoisomerase III and IV activities *in vitro*. Topoisomerase III and IV activities were assayed in the presence of different amounst of TopAI, respectively. The reaction was stopped by the addition of proteinase K and SDS. *Lane 2*, without PrS-TopAI; *lanes 3–6*, 20, 50, 100, and 200 nM PrS-TopAI. (**E**) *E. coli* PLK831(WT) or RS2(*topA* mutant) containing pColdIII or pColdIII-*topAI* was streaked on M9 (glycerol, CAA) plates with or without 0.1 mM IPTG. The plates were incubated at 37°C for 18 h. (**F**) Interaction of TopAI with TopA *in vivo*. Immunoprecipitation was performed as described in Materials and Methods using TopA, TopA67 or TopA14 and PrS-TopAI or PrS (2.5 μg) in the presence or absence of supercoiled DNA, pUC19. Components used for immunoprecipitation are indicated by a plus (+) in the respective row. Immunoprecipitates were then analyzed by western blotting using anti-His-tag monoclonal antibody.

In order to analyze TopAI inhibitory function, topoisomerase I activity was measured in the presence of TopAI *in vitro*. The supercoiled pUC19 plasmid DNA and topoisomerase I was incubated at various concentrations of PrS-TopAI and the reaction was stopped by the addition of EDTA (Figure 4A, lanes 2–8) or SDS and proteinase K (Figure 4A, lanes 2–12). The samples were analyzed in the absence (Figure 4A) or presence (Figure 4B) of chloroquine. When the reaction was stopped by EDTA, the DNA was stacked on the top of the gel in the presence of TopAI (Figure 4B, lanes 4–7), indicating that TopAI formed a complex with TopA-DNA. Notably, TopAI did not bind to *E. coli* genomic DNA indicating that TopAI did not have DNA binding activity (Supplementary Figure S1). Topoisomerase I activity was almost completely blocked at the TopAI concentrations of 100 nM or above (Figure 4B, lanes 7 and 8). Notably, the nicked DNA shown by a black arrow did not increase after incubation with TopAI (Figure 4B, lanes 4–8), suggesting that TopAI inhibited the nicking activity of topoisomerase I. We then tested the effect of YjhQ antitoxin on the TopAI-mediated inhibition of topoisomerase I activity. The addition of PrS_2_-YjhQ rescued topoisomerase I activity in a dose-dependent manner (Figure 4A, lane 8). Since it was shown that topoisomerase III (TopB) and topoisomerase IV (ParC and ParE) also have relaxation activity (12,35), we tested if TopAI inhibits topoisomerase III and IV activities, respectively. Notably, the deletion of *parE* but not *topB* and *parC* is lethal, indicating that *parE* is an essential gene in *E. coli* (36). Topoisomerase III or IV protein was incubated with different amounts of TopAI and the reactions were stopped by the addition of SDS and proteinase K. The topoisomerase III and IV activities were not inhibited in the presence of 200 nM TopAI which was enough to inhibit topoisomerase I activity (Figure 4C and D), indicating that TopAI is a specific inhibitor of topoisomerase I. We also tested the toxicity of TopAI in a *topA* mutant, *E. coli* RS2, using PLK831 as a parent cell, in which the IS1 element insertion mutation results in a partially active truncated protein that contained 8 amino acids encoded by the IS1 element added to the first 789 amino acids of TopA and some mutations in *gyrA* and *gyrB* have been shown to weaken the DNA gyrase activity (25). The *topAI* gene was cloned into an IPTG-inducible pColdIII plasmid (37). *E. coli* PLK831 cells harboring pCold-*topAI* did not form colonies on M9-glycerol-casamino acids agar plates in the presence of IPTG (1 mM) (Figure 4E). However, induction of TopAI in *E. coli* RS2 in the presence of 1 mM IPTG had no effect on cell growth. Based on these results, we concluded that TopAI is an endogenous topoisomerase I specific inhibitor in *E. coli*. TopA nuclease activity has been shown to induce cell death (38) but DNA cleavage was not observed after incubation with TopAI, suggesting that TopAI inhibits the TopA nuclease activity.

### TopAI interacts with DNA-TopA complex

Next, we examined the interaction of TopAI with TopA. Since the crystal structures of the N-terminal region of TopA containing the active site (Tyr 319) and the single strand DNA binding site (TopA67 from residue 1 to 597; 67 kDa) and the C-terminal region containing the double stranded DNA binding site (TopA14 from residue 745 to 865; 14 kDa) of TopA were solved (39,40), we used TopA, TopA67 and TopA14 for the experiments. We purified the His-tagged TopA, TopA67, TopA14, PrS and PrS-TopAI using Ni-columns and examined the interaction in the absence of DNA by immunoprecipitation using anti-PrS antiserum as described in Material and Methods. As shown in Figure 4F, TopA67 was coimmunoprecipitated with PrS-TopAI (Figure 4F, lanes 2 and 8) but TopA14 was not co-immunoprecipitated (Figure 4F, lanes 3 and 9). On the other hand, TopA was only weakly detected as shown in lane 7 in Figure 4F. Since TopAI interacted with TopA-DNA complex as shown in Figure 4A, we further analyzed whether DNA is required for interaction of TopAI with TopA. For this, PrS-TopAI or PrS and TopA or TopA variants were incubated on ice for 10 min and then supercoiled plasmid DNA was added. After 30 min incubation at room temperature, immunoprecipitation was performed. TopA67 was equally detected both in the presence or absence of DNA (Figure 4F, lanes 8 and 11). On the other hand, TopA did co-immunoprecipitate with PrS-TopAI only when DNA was added to the reaction mixture (Figure 4F, lanes 7 and 10). These results show that TopAI binds to the N-terminal region of TopA, and that DNA is absolutely required for the interaction between full-length TopA and TopAI. We concluded that TopAI binds to the TopA-DNA complex, resulting in the inhibition of topoisomerase I activity.

## DISCUSSION

In the present paper, we demonstrated that the *topAI-yjhQ* operon is a new toxin-antitoxin system. In contrast to most of other TA systems in which an antitoxin gene is placed first followed by the gene for its cognate toxin, the first gene in the TopAI-YjhQ operon encodes the toxin, TopAI, followed by the gene for the antitoxin, YjhQ. We concluded that TopAI-YjhQ is a new TA system on the basis of the following facts; (i) TopAI is a novel toxin inhibiting cell growth, (ii) YjhQ functions as the antitoxin for TopAI toxin and (iii) TopAI and YjhQ form a stable complex (Figure 1A and B). It has been reported that palindromic sequences exist in the promoter region in many TA systems and TA complexes and/or antitoxins bind to the palindromic sequences resulting in the suppression of the transcription of the TA systems (1). We also found two palindromic sequences (ACACCGCGGTGT and TGCAcgtttTGCA) in the upstream region of *topAI-yjh*Q (Supplementary Figure S2 A-C). We tested if the TopAI-YjhQ complex and/or YjhQ bind to the dsDNA oligonucleotides containing the palindromic sequences present in the promoter region using the electrophoretic mobility shift assay. However, the DNA binding of the TopAI-YjhQ complex and YjhQ was not detected even when the molar ratio between the protein complex and DNA was increased up to 100:1 (Supplementary Figure S2B and C). It is possible that another transcriptional regulator binds to the palindromic sequences to regulate the expression of TopAI and YjhQ.

As shown in Figure 4A and B, TopAI inhibits topoisomerase I activity. TopAI specifically interacts with the N-terminal region of TopA (TopA67) but not full-TopA in the absence of plasmid DNA, while PrS-TopAI binds to full-TopA in the presence of DNA (Figure 4F). The data suggest that the TopAI binding region of the N-terminal TopA domain of TopA is not accessible to TopAI in the absence of DNA. However when TopA binds to DNA, the structure of TopA is changed exposing the binding site in TopA accessible to TopAI. Then TopAI is assumed to bind to the exposed region in TopA so that TopAI binds to an intermediate state of TopA during the relaxation process of DNA. It is known that the N-terminal domain of TopA is able to conformationally change (41). On the other hand, it is not known if the C-terminal domain is also susceptible to structural change during relaxation reaction. Our data suggest that the TopA dynamically changes its structure involving not only in the N-terminal region but also in the C-terminal region during the relaxation reaction. The study of these structure changes in TopA during the relaxation reaction will give us a new insight into the mechanism of activity of TopA. On the basis of these data, it is possible that either the TopAI binding sites are covered with the C-terminal domain or that the conformational change of the N-terminal domain caused by DNA is required. Although topoisomerases III and IV also relax supercoiled DNA, TopAI did not inhibit their activity *in vitro* (Figure 4C and D). It has been suggested that *topA* is an essential gene since the deletion of the gene is lethal (25). Therefore, we also tested the toxicity of TopAI in a *topA* deletion mutant, *E. coli* RS2, in which mutations in *gyrA* and *gyrB* have been shown to weaken the activity (25). The *topAI* gene was cloned into an IPTG-inducible pColdIII plasmid (37). *E. coli* PLK831 [F-, *gal-25*, *λ^−^*, *ΔtrpE63*, *pyrF287*, *fnr-1*, *IN(rrnD-rrnE)1*, *rpsL195*(strR), *iclR7*(Const), *trpR72*(Am)] cells harboring pCold-*topAI* did not form colonies on M9-glycerol-casamino acids agar plates in the presence of IPTG (1 mM) (Figure 4E). However, induction of TopAI in the *topA10* mutant, *E. coli* RS2 [F-, *gal-25*, *λ^−^*, *topA10*, *pyrF287*, *fnr-1*, *rpsL195*(strR), *iclR7*(Const), *trpR72*(Am)] in the presence of 1 mM IPTG showed no effect on cell growth. Although it was also shown that a *topA* mutant is viable in the presence of topoisomerase III (*topB*) (23,24) or some mutations showed to weaken DNA gyrase activity, as shown in Figure 4C, TopAI has no effect on TopB activity. Since the cellular amount of TopB is very low (42), one can assume that its amount may not be enough to complement the TopA activity after the induction of TopAI resulting in the inhibition of protein synthesis. Based on these results, we concluded that TopAI is an endogenous topoisomerase I specific inhibitor in *E. coli*.

It was shown that TopA nuclease activity induces cell death. The expression of a topoisomerase I mutant which does not cause relaxation but retains the ability to cleave DNA and form a covalent complex has been shown to cause oxidative damage leading to cell death (38,43). Although TopAI caused cell death (Figure 1D), we did not find any increase in the amount of nicked DNA after incubation with TopAI (Figure 4B), indicating that TopAI inhibits DNA nicking activity of TopA. These data predict that TopAI leads to cell death by trapping the TopA-DNA complex without the accumulation of nicked DNA, which appears to be different from the mechanism underlying topoisomerase I mediated cell death. It was reported that phage T4 55.2 protein and Tn5 transposase inhibit topoisomerase I activity (44,45). We also analyzed the effect of TopA overexpression on TopAI toxicity. The overexpression of TopA neutralized TopAI toxicity as shown in Supplementary Figure S3 indicating that inhibition of TopA activity by TopAI caused cell death. Phage T4 55.2 protein binds to DNA and inhibits relaxation of negatively supercoiled DNA. On the other hand, TopAI has no DNA binding activity and strongly inhibits TopA activity. This indicates that the inhibition mechanism of TopA by TopI is different from T4 55.2.

In the present paper, we demonstrated that TopAI inhibits topoisomerase I and thereby DNA replication, RNA synthesis and subsequently cell growth. Interestingly, it has been reported that TopAI and YjhQ are induced after the addition of antibiotics such as kanamycin sulfate (18 fold) and gentamycin (15 fold) using DNA micro arrays (46,47). TopAI inhibits cell growth, which possibly leads to a dormancy state. The bacteria can thus escape any further lethal damages on DNA caused by the antibiotics. This suggests a possibility that TopAI plays an important role in the development to the persistence state of the cells after treatment of the antibiotics *via* inhibition of topoisomerase I. It has been reported that deletion of a single toxin gene in the TA systems encoding RNases (*chpBI-chpBK, relB-relE, mqsR-mqsA, yefM-yoeB, dinJ-yafQ, higB-higA, yafN-yafO, prlF-yhaV, hicA-hicB* or *mazE-mazF*) had no effect on persister formation (48,49), while the deletion of all ten RNase-encoding TA systems resulted in low persister formation, indicating that all these ten RNase-encoding TA systems may be required for persistence in *E. coli* (50). Although TopAI inhibits TopA activity but does not cleave mRNA, the effect of the *topAI-yjhQ* TA system on persister formation may be observed, only if multiple deletion strains of the TA systems are tested.

It has been indicated that supercoiling levels of the genomic DNA influences local DNA structures and can affect gene expression (51,52). The expression of 106 genes has been shown to increase after genomic DNA relaxation, whereas the expression of 200 genes decreased, indicating that supercoiling acts as a second messenger by relaying environmental signals (51,52). A role for supercoiling as a global regulator for the growth of *E. coli* was also proposed on the basis of its effect on transcription initiation at the rRNA promoters (51). TopAI may play an important role in the regulation of DNA topology under some stress conditions by the inhibition of TopA.

## Supplementary Material

SUPPLEMENTARY DATA
