# Supplementary material for: An endogenous protein inhibitor, YjhX (TopAI), for topoisomerase I from Escherichia coli
Source: Nucleic Acids Res. 2015 Nov 8;43(21):10387–96. doi: 10.1093/nar/gkv1197 (PMC4666372; doi:10.1093/nar/gkv1197)
Supplement: SUPPLEMENTARY DATA [file supp_43_21_10387__index.html]

An endogenous protein inhibitor, YjhX (TopAI), for topoisomerase I from Escherichia coli — SUPPLEMENTARY DATA 

# An endogenous protein inhibitor, YjhX (TopAI), for topoisomerase I from *Escherichia coli*

## SUPPLEMENTARY DATA

- SUPPLEMENTARY DATA
