# Supplementary material for: An endogenous protein inhibitor, YjhX (TopAI), for topoisomerase I from Escherichia coli
Source: Nucleic Acids Res. 2015 Nov 8;43(21):10387–96. doi: 10.1093/nar/gkv1197 (PMC4666372; doi:10.1093/nar/gkv1197)
Supplement: SUPPLEMENTARY DATA [file supp_gkv1197_nar-01232-f-2015-File006.pdf]

Supplemental Figures

A

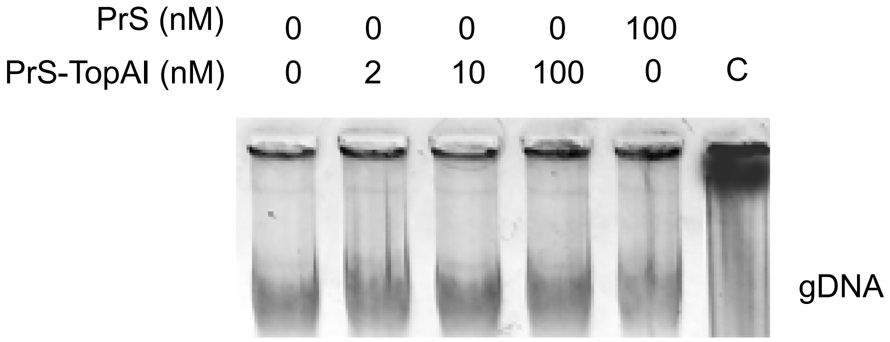

Figure S1. Effect of TopI on DNA *in vitro*. *E. coli* genomic DNA was incubated with different concentrations of purified PrS-TopI in 50 mM Tris-HCl buffer (pH 7.4) containing 1mM EDTA and 0.1 mM DTT, respectively. DpnI (New England lab.) was used as control (shown as C). The DNA was analyzed by 1% agarose gel electrophoresis.

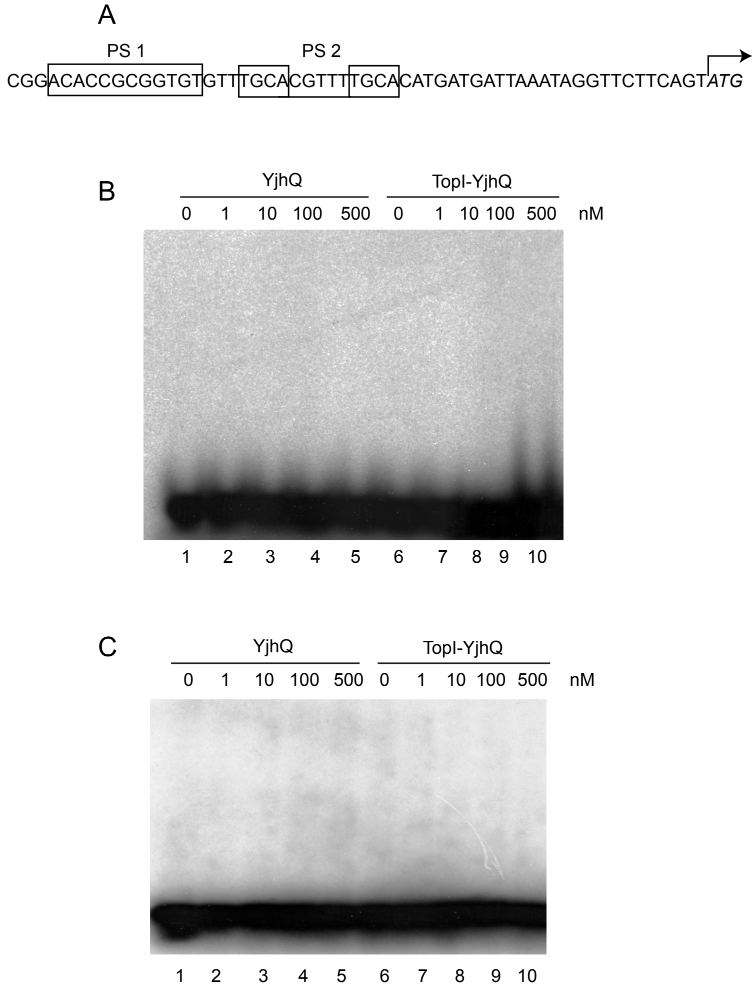

Figure S2. DNA binding assay of YjhQ and the TopI-YjhQ complex to the 5'-UTR region. (**A**) The sequence of the *topI* 5'-UTR region. Two palindromic sequences (PS1 and PS2) are boxed, respectively. The start codon (ATG) is shown in italic. (**B** and **C**) The electrophoretic mobility shift assay (EMSA) was carried out with 5'-end-[^{32}P]labeled oligo DNA, containing PS1 and PS2. The DNA (1 nM) was incubated with the YjhQ or the TopI-YjhQ complex in different molar ratios.

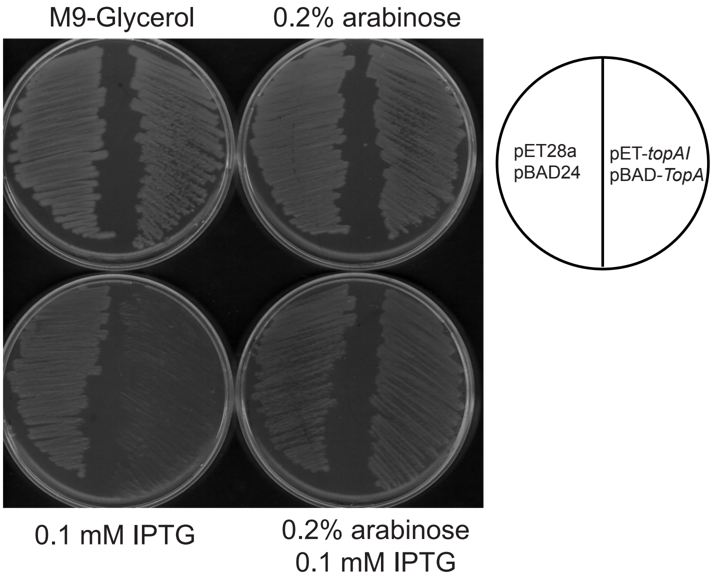

Figure S3. Neutralization of TopAI toxicity with overexpression of TopA. *E. coli* BL21 transformed with pET28a and pBAD24 or pET-*topAI* and pBAD-*topA* was streaked on M9 (glycerol, CAA) plates with 0.1 mM IPTG, 0.2% arabinose, 0.1 mM IPTG plus 0.2% arabinose or without both inducers. The plates were incubated at 37˚C for 18 h.
